# Robotic Hysterectomy with Ureter Identification and Uterine Artery Ligation for Benign Gynecological Conditions: An Early-Year Single-Center Experience

**DOI:** 10.3390/diagnostics13101809

**Published:** 2023-05-20

**Authors:** Yi-Liang Lee, Kai-Jo Chiang, Chi-Kung Lin, Tai-Kuang Chao, Mu-Hsien Yu, Yung-Liang Liu, Yu-Chi Wang

**Affiliations:** 1 Department of Obstetrics and Gynecology, Tri-Service General Hospital, National Defense Medical Center, Taipei 114, Taiwan; lylobgyn@gmail.com (Y.-L.L.);; 2 Department of Obstetrics and Gynecology, Kang-Ning General Hospital, Kang Ning University, Taipei 114, Taiwan; 3 Department of Nursing, Tri-Service General Hospital, National Defense Medical Center, Taipei 114, Taiwan; 4 School of Nursing, National Defense Medical Center, Taipei 114, Taiwan; 5 Department of Pathology, Tri-Service General Hospital, National Defense Medical Center, Taipei 114, Taiwan; 6 Division of Reproductive Medicine, Department of Obstetrics and Gynecology, Chung Shan Medical University Hospital, Taichung 402, Taiwan

**Keywords:** robotic hysterectomy, ureter identification, uterine artery ligation

## Abstract

The use and application of robotic systems with a high-definition, three-dimensional vision system and advanced EndoWrist technology have become widespread. We sought to share our clinical experience with ureter identification and preventive uterine artery ligation in robotic hysterectomy. The records of patients undergoing robotic hysterectomy between May 2014 and December 2015, including patient preoperative characteristics, operative time, and postoperative outcomes, were analyzed. We evaluated the feasibility and safety of using early ureteral identification and preventive uterine artery ligation in robotic hysterectomy in patients with benign gynecological conditions. Overall, 49 patients diagnosed with benign gynecological conditions were evaluated. The mean age of the patients and mean uterine weight were 46.2 ± 5.3 years and 348.7 ± 311.8 g, respectively. Robotic hysterectomy achieved satisfactory results, including a short postoperative hospital stay (2.7 ± 0.8 days), low conversion rate (n = 0), and low complication rate (n = 1; 2%). The average estimated blood loss was 109 ± 107.2 mL. Our results suggest that robotic hysterectomy using early ureteral identification and preventive uterine artery ligation is feasible and safe in patients with benign gynecological conditions.

## 1. Introduction

Over the years, laparoscopic surgery has been increasingly adopted by gynecologic surgeons due to its numerous advantages over laparotomy, including shorter hospital stays, faster recoveries, fewer postoperative complications, and better aesthetic outcomes. As technology advances, minimally invasive surgery in gynecology continues to evolve, enabling gynecologic surgeons to provide improved patient care. In 2005, the da Vinci robotic surgical system was approved by the U.S. Food and Drug Administration for use in gynecologic surgery and other surgical fields such as urology, orthopedics, general surgery, and cardio-thoracic surgery [1,2,3,4,5,6,7,8,9,10]. Robotic surgical systems equipped with EndoWrist technology and a high-definition, three-dimensional vision system have been implemented in gynecologic surgery to address the limitations and drawbacks of traditional laparoscopy. These systems provide surgeons with increased precision and dexterity, allowing them to perform complex surgical maneuvers with greater ease and efficiency. The use of robotic systems has the potential to lead to reduced complications, minimized blood loss, and shorter hospitalization times for patients. However, the implementation of robotic systems requires a significant investment and specialized training to ensure effective utilization, in addition to the high acquisition and maintenance costs. Despite these challenges, the benefits of robotic systems may ultimately establish them as a standard option for gynecologic surgical procedures [11]. From a medical perspective, the use of robotic technology has led to increased accuracy and precision, reduced complications, shorter recovery times, and improved patient outcomes. These advancements have ultimately led to a paradigm shift in the field of surgery, paving the way for more minimally invasive and efficient surgical techniques [8]. The use of robotically assisted hysterectomy in complex cases, including malignancies, large uteri, obesity, and severe adhesions, has been shown to be feasible [12,13]. In a nationwide database study, the effect of robotic technology on benign hysterectomy procedures and clinical outcomes was investigated. The study reviewed data from four main approaches to hysterectomy: abdominal, vaginal, laparoscopic, and robotic. The results demonstrated that robotic hysterectomy was a successful and safe approach, leading to a rise in the use of minimally invasive surgeries, without compromising patient outcomes. These findings further emphasize the benefits of robotic technology in the field of gynecology [14]. An earlier publication by our team presented the first descriptive series of robotic surgery in Taiwan, specifically for patients with complex gynecologic conditions. The study demonstrated that robotic surgery was both safe and feasible, with positive outcomes for the patients involved. The success of this study further highlights the advantages of robotic technology in the field of gynecology [7].

Based on the findings of previous studies regarding iatrogenic injuries during gynecologic and obstetric operations, it is imperative for medical professionals to be aware of the potential occurrence of ureteral injury during such procedures. Notably, such injuries can lead to severe morbidity and have serious complications, underscoring the significance of maintaining vigilance and employing proper surgical techniques to mitigate the risk of such injuries [15]. The incidence is approximately 0.2 and 1.3 per 1000 cases of vaginal and abdominal hysterectomies, respectively [16]. Iatrogenic ureteral injuries are a common occurrence during gynecologic surgeries, with the pelvic ureter being the most susceptible segment. However, timely identification and proper management of such injuries can prevent or minimize renal function loss. This emphasizes the importance of exercising caution during pelvic surgeries and remaining alert for potential signs of ureteral injury, including fever or flank pain, which should prompt immediate evaluation with imaging studies [17]. 

In light of advanced vision system technologies in robotic surgical systems, the pelvic ureter can be visualized with adequate precision during a variety of gynecologic surgeries. The current study examines our experience in performing robotic hysterectomy with early ureteral identification and preventive uterine artery ligation, assessing both the feasibility of this approach and potential pitfalls and challenges that may arise. This study aims to emphasize the importance of meticulous surgical planning and technique execution to reduce the risk of both ureteral injury and blood loss during gynecologic surgery, particularly in cases involving robotic hysterectomy.

## 2. Materials and Methods

Forty-nine patients underwent robotic subtotal hysterectomy between May 2014 and December 2015 at a tertiary medical center in northern Taiwan. The patients included in the study were consecutive cases during the study period. Of these, 23 patients were diagnosed with benign gynecological disease, 23 were diagnosed with leiomyomas of the uterus, 11 were diagnosed with adenomyosis of the uterus, and 15 were diagnosed with leiomyomas and adenomyosis of the uterus (Table 1). All patients underwent surgery consecutively using the da Vinci Si robotic surgical system (Intuitive Surgical Inc., Sunnyvale, CA, USA) at our institution. Preoperative characteristics and postoperative data of the 49 patients were screened. All surgical procedures were performed by a single surgeon and rotating assistants, according to the procedure described in the following section. This study was conducted in accordance with the principles of the Declaration of Helsinki and approved by the Institutional Review Board (IRB) of the Tri-Service General Hospital (TSGHIRB No. 1-104-05-152).

Patients were placed in the Trendelenburg position while under general anesthesia. A urinary catheter and a uterine manipulator were placed before the surgery. After pneumoperitoneum was created through CO_2_ insufflation pressure using a transumbilical Veress needle, four trocars were placed in the patients’ abdomen: one 12 mm central port, two 8 mm ports for the robotic arms, and one additional 12 mm or 5 mm port for the assistant. Following the docking of the robotic arms, the three-dimensional 0- or 30-degree stereoscopic endoscope and EndoWrist instruments were inserted into the robotic ports. Robotic surgery was performed with EndoWrist instruments, including PreCise bipolar forceps, and a monopolar cautery spatula with or without a large/mega needle driver (Intuitive Surgical Inc.). A grasper or curved scissors were used for assistance via an accessory port. The surgeon then went to the console and controlled the robot remotely. After identifying each side of the ureter, ligation of the same side of the uterine artery and adhesiolysis were performed. The main procedures and a survey of the operative field for hemostasis were then performed. The arms were undocked, and the instruments were removed. The endobag with the hand morcellation method was utilized to remove the specimen through the trocar site. The survey of the operative field for hemostasis was performed again. Finally, the intra-abdominal gas was released, and the trocar sites were closed with sutures proximate to the fascia and subcutaneous tissues. Critical time intervals, including the docking time, console time, and total operative time, have been described previously [4].

## 3. Results

In total, 49 patients underwent subtotal hysterectomy, with an average age of 46.2 ± 5.3 years, and a mean body mass index of 23.2 ± 3.0 kg/m^2^. Of these patients, 67.35% had a history of previous abdominal surgery, which could potentially impact the surgical outcomes in these patients (Table 1). 

### Ureter Identification and Ligation of Uterine Arteries

**Figure 1:**
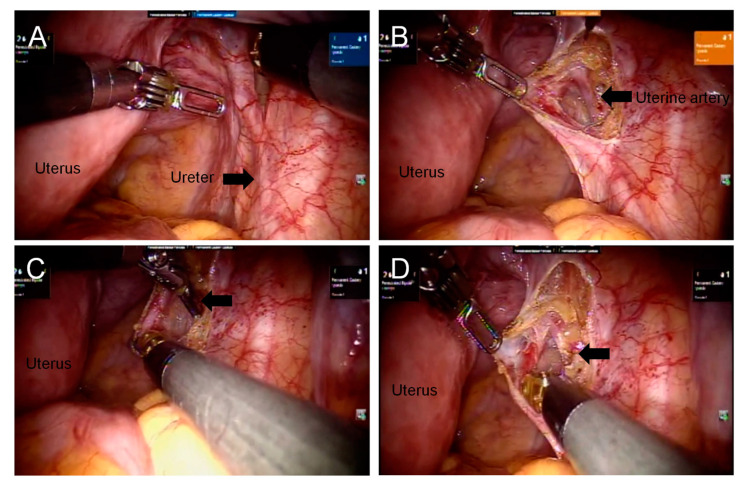
Visualization of the right ureter and ligation of the right uterine artery. (**A**) The right ureter (arrow) can be visualized at the level of the pelvic brim. (**B**) Blunt dissection of the right ureter was carried out using dissecting forceps, extending from its peritoneal attachment down to its course to the parametrial level. Subsequently, the right uterine artery (indicated by an arrow) was identified and coagulated using bipolar forceps. (**C**) The right uterine artery was coagulated using bipolar forceps (arrow). (**D**) The completed ligation of the right uterine artery (arrow).

**Figure 2:**
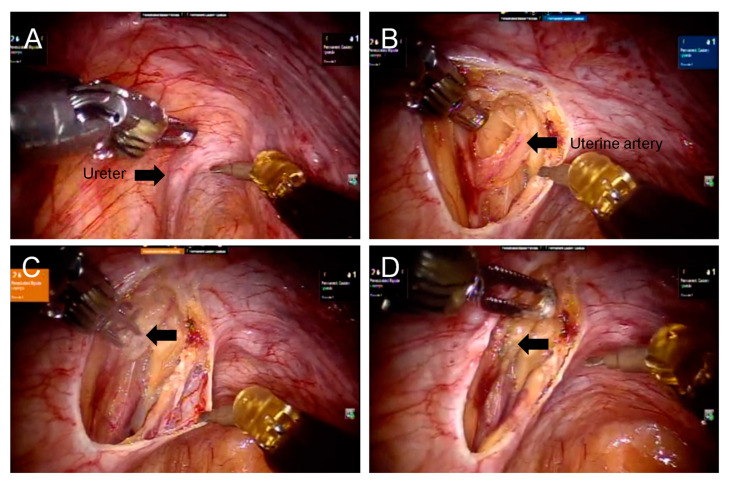
Visualization of the left ureter and ligation of the left uterine artery. (**A**) The left ureter (arrow) can be visualized at the level of the pelvic brim. (**B**) The left ureter was dissected bluntly using dissecting forceps from its peritoneal attachment down to its course to the parametrial level. The left uterine artery (arrow) was then identified and coagulated using bipolar forceps. (**C**) The left uterine artery was coagulated using bipolar forceps (arrow). (**D**) The completed ligation of the left uterine artery (arrow).

With advanced vision system technologies in the robotic surgical system, the pelvic ureters were adequately visualized at the level of the pelvic brim and along the lateral pelvic peritoneum in most patients during hysterectomy (Figure 1A and Figure 2A). Upon initial exploration, both the right and left ureters were identified, and subsequently, the peritoneum was opened. A careful dissection of the ureters was performed in a caudal direction, along the pelvic peritoneum, to allow for observation of the uterine artery originating from the internal iliac artery (Figure 1B and Figure 2B). In order to further identify the uterine artery, we traced the umbilical ligament. Once the uterine arteries were located, they were ligated bilaterally using electrocauterization (Figure 1C,D and Figure 2C,D). After further dissection of the ureters from the cervix, hysterectomy was performed in the supracervical region. The mean docking time was 11 ± 7 min, the mean console time was 91.1 ± 36.7 min, and the mean total operative time was 134.8 ± 57.5 min (Table 2). The average uterus weight was 348.7 ± 311.8 g, and the maximum uterine weight was 1320 g. The mean preoperative and postoperative hemoglobin (Hgb) levels were 10.8 ± 2.8 g/dL and 9.5 ± 2.3 g/dL, respectively. The mean estimated intraoperative blood loss was 109.1 ± 107.2 mL. Seven (14.3%) patients received a blood transfusion either during or after the surgery. The mean postoperative hospital stay was 2.7 ± 0.8 days. It should be noted that there were no occurrences of conversion to mini-laparotomy or conventional laparotomy in any of the patients who underwent the procedure. Additionally, most patients had an unremarkable postoperative course. A small bowel serosal tear was noted in one (2%) patient, presenting with severe lower abdominal pain and intestinal fluid-like drainage during hospitalization (Table 3). The general surgeon repaired this small bowel serosal tear using laparoscopy. She recovered uneventfully during the outpatient clinic follow-up.

## 4. Discussion

In this study, our results demonstrated the feasibility and outcomes of robotic hysterectomy with early ureteral identification and preventive uterine artery ligation. Our findings suggest that this technique allows for safe and effective robotic hysterectomy, even in challenging situations, such as cases involving a maximum uterine weight of 1320 g or less. This study emphasizes the importance of careful patient selection, preoperative planning, and surgical skill in minimizing the risk of complications, including ureteral injury. These findings are in line with a recent pilot study that developed a risk assessment model for complications in minimally invasive hysterectomy, which included predictors such as BMI, previous surgery, and the surgeon’s experience [18]. Additionally, this study highlights the benefits of robotic surgery, such as improved visualization and dexterity, which may contribute to the success of this approach. With the assistance of advanced three-dimensional vision system technologies and EndoWrist instruments, we can approach ureters and ligate uterine arteries effectively to prevent ureter injuries and diminish blood loss during robotic hysterectomy. 

Ureter injuries are one of the complications surgeons are most apprehensive of when performing hysterectomies [15]. The incidence of ureteral injuries during laparoscopic hysterectomy may be attributed to a range of predisposing factors, including inadequate training or experience of the surgeon in the technique, insufficient application of surgical skills such as coagulation of uterine arteries without the use of a uterine manipulator, and/or failure to perform ureterolysis in patients with a distorted anatomy. Studies conducted in the Netherlands and France have reported on the causes and prevention of laparoscopic ureteral injuries, highlighting the importance of surgeon experience and a careful surgical technique in minimizing the risk of complications [19,20]. To prevent ureter injuries during laparoscopic hysterectomy, it is recommended to use uterine manipulators for a better overview of the anatomy [21]. The insertion of ureter stents before surgery could be considered in cases of suspected distorted anatomy, such as deep infiltrating endometriosis (DIE) [21,22]. One study focusing on ureter injuries in gynecological and obstetrical surgery from completed insurance claims revealed that most injuries could be avoided by adequately exposing the ureter via dissection [23]. Despite the fine dexterity and three-dimensional vision afforded by the robotic instruments, ureter injuries have also been reported during robotic surgery in patients undergoing hysterectomy [24] and DIE [25]. We routinely used a uterine manipulator to obtain a better overview of the anatomy and exposed the ureters adequately via dissection with advanced vision system technologies in the robotic surgical system during robotic hysterectomy. The incidence of ureter injuries may decrease thereafter. 

Previous reports have described how to perform laparoscopic uterine artery ligation [26,27,28,29,30,31]. These include the anterior approach, through the anterior board ligament; the posterior approach, through the posterior broad ligament; the lateral approach, through a peritoneum lateral to the infundibulopelvic ligament; the retrograde umbilical ligament approach; or an early ureteral identification technique. We used an early ureteral identification approach. By dissecting the course of the ureter, caudally, we isolated and identified the uterine artery. The uterine artery was then ligated via electrocauterization. We utilized a modified technique of uterine artery ligation to achieve hemostasis during the procedure. By carefully identifying and ligating the branches of the uterine artery, we were able to decrease blood flow to the uterus and reduce the risk of hemorrhage. Our approach may result in a significant reduction in blood loss and transfusion rates during the surgery.

Hysterectomy, the surgical removal of the uterus, is a commonly performed procedure that can be accomplished through various techniques, including vaginal, abdominal, laparoscopic, robot-assisted laparoscopic, or hybrid methods, such as laparoscopy-assisted transvaginal hysterectomy. In a 2015 Cochrane systematic review and meta-analysis of 47 studies comprising 5102 women undergoing hysterectomy for benign gynecological disease, various surgical approaches including abdominal, vaginal, laparoscopic, and robotic-assisted hysterectomy were compared. The authors concluded that in cases where it is technically feasible, vaginal hysterectomy should be considered as the first-line option. Laparoscopic hysterectomy may replace abdominal hysterectomy when vaginal hysterectomy is not possible; however, it is associated with a higher incidence of urinary tract injuries. Notably, there is a lack of strong evidence supporting the use of robotic-assisted hysterectomy in reducing injuries during the procedure [32]. In a 2016 systematic review and meta-analysis of randomized trials comparing robotic versus laparoscopic hysterectomy for benign disease in 326 women, the authors found no significant differences in complication rates. The study also showed that other important outcomes, such as the length of hospital stay, total operating time, conversion to laparotomy, and blood loss, were similar between the two procedures. It is important to note, however, that this study is limited to the specific population of women with benign disease and may not be applicable to other patient populations. Therefore, it is recommended to consult with a healthcare provider to determine the most appropriate treatment approach for each individual patient [33]. Despite the growing body of literature indicating the limited benefits of robotic surgery in patients with benign gynecologic conditions, our study findings suggest that robotic hysterectomy is technically feasible with acceptable outcomes in terms of the operative time, estimated blood loss, transfusion rates, and postoperative stay. While the results of our study are encouraging, further investigations are warranted to better elucidate the role of robotic surgery in this patient population. Future studies may help to identify which patient subgroups might benefit the most from robotic-assisted hysterectomy and the potential advantages of this approach over conventional surgical methods. Additionally, such research could help to guide the development of appropriate patient selection criteria and optimal surgical techniques, thereby improving the overall quality of care for women undergoing hysterectomy for benign gynecological conditions. Studies have demonstrated that implementing best practices through standardized care is linked to improved outcomes and reduced costs [34].

In our research, we found that out of the 49 patients, only 7 (14.3%) needed a blood transfusion, which is a higher rate compared to previous studies on robotic hysterectomy, ranging from 0.1% to 9.7% [13]. The increased transfusion rate could be attributed to the anesthesiology preoperative requirement, which mandates blood transfusion for patients with a preoperative Hgb < 7. Additionally, three patients required postoperative blood transfusion due to a postoperative Hgb < 9. However, it is crucial to note that transfusion decisions must be made on a case-by-case basis, considering the patient’s medical history, hemodynamic status, and the extent of blood loss.

In the realm of gynecology, subtotal hysterectomy has emerged as a viable alternative for women with benign gynecologic conditions who wish to retain their cervix. To delve deeper into the benefits of this surgical procedure, a 2012 meta-analysis was conducted, involving nine trials with a total of 1553 women. The study’s findings revealed that major outcomes, including urinary, bowel, or sexual function, did not differ significantly between women who underwent total hysterectomy and those who underwent subtotal hysterectomy. Furthermore, other outcomes such as blood transfusion, complications, recovery, and alleviation of pre-surgery symptoms were found to be comparable in both groups. However, cyclic vaginal bleeding was more common among women who had undergone subtotal hysterectomy, as compared to those who had a total hysterectomy [35]. In our study, the majority of patients were subjected to robotic subtotal hysterectomy, during which we informed them about the need for cancer screening and the possibility of experiencing cyclic vaginal bleeding. While the benefits of subtotal hysterectomy remain a matter of debate, our findings indicate that robotic hysterectomy can be conducted with minimal risk to the patient.

## 5. Conclusions

In conclusion, our study adds to the growing body of evidence supporting the safety and feasibility of robotic hysterectomy for benign gynecologic conditions. Our results showed favorable surgical outcomes, including minimal blood loss, a low transfusion rate, a short hospital stay, and few complications. Patients who underwent robotic subtotal hysterectomy had high satisfaction rates and minimal postoperative pain, with no need for conversion to conventional laparotomy. Moreover, we demonstrated the effectiveness of a preventive technique to identify and ligate the uterine artery, which can minimize the risks. Despite some limitations, such as the small sample size and lack of long-term follow-up data, our findings indicate that robotic hysterectomy with this technique can improve patient outcomes and reduce healthcare costs. Further research is necessary to determine the role and potential drawbacks of robotic surgery and to establish clear guidelines for patient selection.

## Figures and Tables

**Table 1:** Patient characteristics.

Age (y)	46.2 ± 5.3
Body mass index (kg/m^2^)	23.2 ± 3.0
History of abdominal surgery	67.35%
Leiomyomas of uterus, n (%)	23 (46.9%)
Adenomyosis of uterus, n (%)	11 (22.4%)
Leiomyomas and adenomyosis of uterus, n (%)	15 (30.6%)

Data are shown as the mean ± standard deviation.

**Table 2:** Robotic surgical details.

Docking time, min	11 ± 7
Console time, min	91.1 ± 36.7
Total operative time (skin to skin), min	134.8 ± 57.5

Data are shown as the mean ± standard deviation.

**Table 3:** Surgical outcomes.

Uterus weight (g)	348.7 ± 311.8
Preoperative Hgb	10.8 ± 2.8
Postoperative Hgb	9.5 ± 2.3
Preoperative Hgb–Postoperative Hgb	1.1 ± 1.2
Estimated blood loss (mL)	109.1 ± 107.2
Transfusion rates, n (%)	7 (14.3%)
Postoperative hospital stay (d)	2.7 ± 0.8
Conversion rate, n (%)	0
Complication rate, n (%)	1 (2%)

Data are shown as the mean ± standard deviation.

## Data Availability

Not applicable.

## References

[1] Advincula A.P., Wang K. (2009). Evolving Role and Current State of Robotics in Minimally Invasive Gynecologic Surgery. J. Minim. Invasive Gynecol..

[2] Chen S.-H., Li Z.-A., Huang R., Xue H.-Q. (2016). Robot-assisted versus conventional laparoscopic surgery for endometrial cancer staging: A meta-analysis. Taiwan. J. Obstet. Gynecol..

[3] Lee C.-L., Wu K.-Y., Su H., Han C.-M., Huang C.-Y., Yen C.-F. (2015). Robot-assisted natural orifice transluminal endoscopic surgery for hysterectomy. Taiwan. J. Obstet. Gynecol..

[4] Cheng H.Y., Chen Y.J., Wang P.H., Tsai H.W., Chang Y.H., Twu N.F., Juang C.-M., Huahsi W., Ming-Shyen Y., Kuan-Chong C. (2015). Robotic-assisted laparoscopic complex myomectomy: A single medical center’s experience. Taiwan J. Obstet. Gynecol..

[5] Huang H.-H., Liu L.-C., Tan S.-J., Lai H.-C. (2013). Robot-assisted excision of a symptomatic deep infiltrating cervical endometrioma. Taiwan. J. Obstet. Gynecol..

[6] Göçmen A., Şanlıkan F., Uçar M.G. (2013). Robot-assisted tubal reanastomosis: Initial experience in a single institution. Taiwan. J. Obstet. Gynecol..

[7] Tan S.-J., Lin C.-K., Fu P.-T., Liu Y.-L., Sun C.-C., Chang C.-C., Yu M.-H., Lai H.-C. (2012). Robotic surgery in complicated gynecologic diseases: Experience of Tri-Service General Hospital in Taiwan. Taiwan. J. Obstet. Gynecol..

[8] Lee C.-L., Han C.-M., Su H., Wu K.-Y., Wang C.-J., Yen C.-F. (2010). Robot-assisted Laparoscopic Staging Surgery for Endometrial Cancer—A Preliminary Report. Taiwan. J. Obstet. Gynecol..

[9] Mao S.-P., Lai H.-C., Chang F.-W., Yu M.-H., Chang C.-C. (2007). Laparoscopy-assisted Robotic Myomectomy Using the DA Vinci System. Taiwan. J. Obstet. Gynecol..

[10] Wang Y.-C., Bai M.-Y., Hsu W.-Y., Yu M.-H. (2018). Evaluation of a series of silk fibroin protein-based nonwoven mats for use as an anti-adhesion patch for wound management in robotic surgery. J. Biomed. Mater. Res. Part A.

[11] Nezhat C., Lavie O., Lemyre M., Unal E., Nezhat C.H., Nezhat F. (2009). Robot-assisted laparoscopic surgery in gynecology: Scientific dream or reality?. Fertil Steril..

[12] Advincula A.P., Reynolds R.K. (2005). The Use of Robot-Assisted Laparoscopic Hysterectomy in the Patient With a Scarred or Obliterated Anterior Cul-de-sac. JSLS J. Soc. Laparosc. Robot. Surg..

[13] Boggess J.F., Gehrig P.A., Cantrell L., Shafer A., Mendivil A., Rossi E., Hanna R. (2009). Perioperative Outcomes of Robotically Assisted Hysterectomy for Benign Cases with Complex Pathology. Obstet. Gynecol..

[14] Luciano A.A., Luciano D.E., Gabbert J., Seshadri-Kreaden U. (2016). The impact of robotics on the mode of benign hysterectomy and clinical outcomes. Int. J. Med. Robot. Comput. Assist. Surg..

[15] Neuman M., Eidelman A., Langer R., Golan A., Bukovsky I., Caspi E. (1991). Iatrogenic injuries to the ureter during gynecologic and obstetric operations. Surg. Gynecol. Obstet..

[16] Burks F.N., Santucci R.A. (2014). Management of iatrogenic ureteral injury. Ther. Adv. Urol..

[17] Selzman A.A., Spirnak J.P. (1996). Iatrogenic Ureteral Injuries: A 20-Year Experience in Treating 165 Injuries. J. Urol..

[18] Bruno M., Legge F., Gentile C., Carone V., Stabile G., Di Leo F., Ludovisi M., Di Florio C., Guido M. (2022). Risk Assessment Model for Complications in Minimally Invasive Hysterectomy: A Pilot Study. Int. J. Environ. Res. Public Health.

[19] Janssen P.F., Brölmann H.A.M., Huirne J.A.F. (2013). Causes and prevention of laparoscopic ureter injuries: An analysis of 31 cases during laparoscopic hysterectomy in the Netherlands. Surg. Endosc..

[20] Leonard F., Fotso A., Borghese B., Chopin N., Foulot H., Chapron C. (2007). Ureteral complications from laparoscopic hysterectomy indicated for benign uterine pathologies: A 13-year experience in a continuous series of 1300 patients. Hum. Reprod..

[21] Sandberg E.M., Hehenkamp W.J.K., Geomini P.M., Janssen P.F., Jansen F.W., Twijnstra A.R.H. (2017). Laparoscopic hysterectomy for benign indications: Clinical practice guideline. Arch. Gynecol. Obstet..

[22] Redan J.A., McCarus S.D. (2009). Protect the ureters. JSLS.

[23] Hove L.D., Bock J., Christoffersen J.K., Andreasson B. (2010). Analysis of 136 ureteral injuries in gynecological and obstetrical surgery from completed insurance claims. Acta Obstet. Gynecol. Scand..

[24] Menderes G., Clark L.E., Azodi M. (2015). Incidental Ureteral Injury and Repair during Robotic-assisted Total Laparoscopic Hysterectomy. J. Minim. Invasive Gynecol..

[25] Collinet P., Leguevaque P., Neme R.M., Cela V., Barton-Smith P., Hébert T., Hanssens S., Nishi H., Nisolle M. (2014). Robot-assisted laparoscopy for deep infiltrating endometriosis: International multicentric retrospective study. Surg. Endosc..

[26] Alborzi S., Ghannadan E., Alborzi S., Alborzi M. (2009). A comparison of combined laparoscopic uterine artery ligation and myomectomy versus laparoscopic myomectomy in treatment of symptomatic myoma. Fertil Steril..

[27] Chang W.-C., Torng P.-L., Huang S.-C., Sheu B.-C., Hsu W.-C., Chen R.-J., Chow S.-N., Chang D.-Y. (2005). Laparoscopic-assisted vaginal hysterectomy with uterine artery ligation through retrograde umbilical ligament tracking. J. Minim. Invasive Gynecol..

[28] Gol M., Kizilyar A., Eminoglu M. (2007). Laparoscopic hysterectomy with retroperitoneal uterine artery sealing using LigaSure™: Gazi hospital experience. Arch. Gynecol. Obstet..

[29] Liu W.-M., Tzeng C.-R., Yi-Jen C., Wang P.-H. (2004). Combining the uterine depletion procedure and myomectomy may be useful for treating symptomatic fibroids. Fertil. Steril..

[30] Aust T., Reyftmann L., Rosen D., Cario G., Chou D. (2011). Anterior Approach to Laparoscopic Uterine Artery Ligation. J. Minim. Invasive Gynecol..

[31] Kobayashi E., Nagase T., Fujiwara K., Hada T., Ota Y., Takaki Y., Kanao H., Andou M. (2012). Total laparoscopic hysterectomy in 1253 patients using an early ureteral identification technique. J. Obstet. Gynaecol. Res..

[32] Aarts J.W., Nieboer E.T., Johnson N., Tavender E., Garry R., Mol B.W.J., Kluivers K.B. (2015). Surgical approach to hysterectomy for benign gynaecological disease. Cochrane Database Syst. Rev..

[33] Albright B.B., Witte T., Tofte A.N., Chou J., Black J.D., Desai V.B., Erekson E.A. (2016). Robotic Versus Laparoscopic Hysterectomy for Benign Disease: A Systematic Review and Meta-Analysis of Randomized Trials. J. Minim. Invasive Gynecol..

[34] Govaert J.A., van Dijk W.A., Fiocco M., Scheffer A.C., Gietelink L., Wouters M.W., Tollenaar R.A. (2016). Nationwide Outcomes Measurement in Colorectal Cancer Surgery: Improving Quality and Reducing Costs. J. Am. Coll. Surg..

[35] Lethaby A., Mukhopadhyay A., Naik R. (2012). Total versus subtotal hysterectomy for benign gynaecological conditions. Cochrane Database Syst. Rev..

